# Influence of different intraoperative fluid management on postoperative outcome after abdominal tumours resection

**DOI:** 10.2478/raon-2024-0015

**Published:** 2024-03-07

**Authors:** Matej Jenko, Katarina Mencin, Vesna Novak-Jankovic, Alenka Spindler-Vesel

**Affiliations:** Department of Anesthesiology and Surgical Intensive Care, University Medical Centre Ljubljana, Slovenia; Faculty of Medicine, University of Ljubljana, Ljubljana, Slovenia

**Keywords:** postoperative complications, intraoperative monitoring, multimodal monitoring, hemodynamic monitoring, cerebral tissue oxygenation, abdominal surgery

## Abstract

**Background:**

Intraoperative fluid management is a crucial aspect of cancer surgery, including colorectal surgery and pancreatoduodenectomy. The study tests if intraoperative multimodal monitoring reduces postoperative morbidity and duration of hospitalisation in patients undergoing major abdominal surgery treated by the same anaesthetic protocols with epidural analgesia.

**Patients and methods:**

A prospective study was conducted in 2 parallel groups. High-risk surgical patients undergoing major abdominal surgery were randomly selected in the control group (CG), where standard monitoring was applied (44 patients), and the protocol group (PG), where cerebral oxygenation and extended hemodynamic monitoring were used with the protocol for intraoperative interventions (44 patients).

**Results:**

There were no differences in the median length of hospital stay, CG 9 days (interquartile range [IQR] 8 days), PG 9 (5.5), p = 0.851. There was no difference in postoperative renal of cardiac impairment. Procalcitonin was significantly higher (highest postoperative value in the first 3 days) in CG, 0.75 mcg/L (IQR 3.19 mcg/L), than in PG, 0.3 mcg/L (0.88 mcg/L), p = 0.001. PG patients received a larger volume of intraoperative fluid; median intraoperative fluid balance +1300 ml (IQR 1063 ml) than CG; +375 ml (IQR 438 ml), p < 0.001.

**Conclusions:**

There were significant differences in intraoperative fluid management and vasopressor use. The median postoperative value of procalcitonin was significantly higher in CG, suggesting differences in immune response to tissue trauma in different intraoperative fluid status, but there was no difference in postoperative morbidity or hospital stay.

## Introduction

Intraoperative fluid management is a crucial aspect of cancer surgery, as it may significantly impact patient outcomes and postoperative complications. The optimal approach to fluid therapy during cancer surgery remains a topic of debate and ongoing research. Several studies have investigated the effects of different fluid management strategies on morbidity, mortality, and postoperative complications in various types of cancer surgeries. Restrictive fluid management has been shown to be superior to standard fluid management in preventing postoperative complications in abdominal surgery. Additionally, goal-directed fluid therapy targeting hemodynamic variables such as cardiac output and stroke volume has been found to decrease fluid balance and reduce inflammatory reactions after lung cancer surgery.^1^

It is important to note that the management of fluid balance in cancer surgery is complex and depends on various factors such as the type of surgery, patient characteristics, and underlying conditions. The use of enhanced recovery after surgery (ERAS) protocols, which include specific guidelines for perioperative fluid management, has been recommended in oncology surgeries.^2^ Outcome of treatment has often been influenced by several variables.^3^

Continuous intraoperative measurement of blood flow and related variables was studied several times to show the benefit for patients. New monitors and treatment protocols with predefined treatment limits (goal-directed optimization of hemodynamic parameters) suggested an improvement in long-term patient outcome and a reduction in morbidity and mortality of more than 50% in some studies.^4, 5, 6, 7^ They aim to optimise microcirculation and improve oxygen delivery by correcting specific hemodynamic parameters.^8^ The benefit of personalised and targeted oxygen delivery algorithms that incorporate both fluid resuscitation and vasoactive drugs applied to high-risk surgical patients was shown.^9^ However, flow monitoring alone when added to conventional monitoring has much less effect on improving outcomes and reducing mortality than anticipated.^10,11^ When using this strategy there was no decrease in mortality and the length of stay decreased on average by only one day.^10^ Also a composite outcome of complications or mortality at 30 days is not reduced by this strategy.^11^ In addition to hemodynamic variables, other important parameters, such as regional cerebral oxygenation (rSO2), measured by near-infrared spectroscopy (NIRS), should be continuously monitored to improve outcomes. Especially in the elderly, the reduction of regional cerebral oxygenation can lead to a poor outcome.^12, 13, 14^ Monitors that assess the degree of cortical suppression (e.g. BIS, Aspect Medical Systems, Cambridge, USA) facilitate anaesthetic titration and have been shown to reduce anaesthetic exposure.^15,16^

In most studies, all new methods have been studied separately, and there is a lack of studies showing the effect of joint (multimodal) monitoring on mortality and occurrence of complications. All data collected indicate that the combined use of new methods (monitoring blood flow with assessment of fluid status, depth of anaesthesia and tissue oxygenation) with adherence to an appropriate protocol could dramatically improve perioperative management and outcome of high-risk surgical patients.^17,18^ The important cofactor that may interfere with the results of the studies is the different anaesthesia techniques used in the patients included in the studies (presence or absence of an epidural catheter, different anaesthetic techniques used).^19, 20, 21^

The present study tests the hypothesis that intraoperative multimodal monitoring with hemodynamic optimisation and maintenance of optimal cerebral oxygenation reduces the rate of postoperative complications. Furthermore, multimodal monitoring can reduce the duration of hospitalisation in patients undergoing major abdominal surgery. To minimise bias, all patients in both groups have received the same intraoperative anaesthetic technique with epidural postoperative analgesia and all patients underwent similar gastrointestinal surgical procedures.

## Patients and methods

A prospective randomised trial with 2 parallel groups was conducted at the University Medical Centre (UMC) Ljubljana in years 2015–2018. Patients from the Clinical Department of Abdominal Surgery were included in the study. Adult patients who underwent one of the following major abdominal cancer surgeries were included: stomach surgery, pancreatic surgery, and large intestinal resections. Only high-risk surgical patients, defined as American Society of Anaesthesia (ASA) class 2 or 3 with P-Possum predicted mortality >4% fulfilled criteria for inclusion.^22^ ASA physical status classification system class 2 are patients with mild systemic disease, while class 3 patients are patients with a severe systemic disease that is not life-threatening.^23^ P-possum is Physiological and Operative Severity Score for the enumeration of Mortality and morbidity. With the result, we are able to predict perioperative mortality.^24^ Exclusion criteria were underage, pregnant women, laparoscopic surgery, and palliative procedures.

The study was approved by the Slovenian National Medical Ethics Committee. It was registered with ClinicalTrials.gov, Surgical Outcome and Multimodal Monitoring (SOMM) Identifier: NCT02293473. The article has previously unpublished data from the study.

Power analysis was performed using simulation of results with the Mann-Whitney U test. For a 2-day difference in stay length, with power 0.8 and significance level 0.05, 16 patients in each group are needed. To show the difference in LOS at one day, 40 patients in each group are needed. The calculations are based on a small pilot study with 12 patients in each group. The expected Cohen value -d is 0.660 for a difference of 1 day in length of stay. We have slightly increased the number of patients recruited due to expected loss during follow-up.

All patients scheduled for abdominal cancer surgery were visited by a member of our team a day prior to surgery to obtain informed consent and to answer any questions. Before anaesthesia, patients were randomly assigned into two groups using covariate adaptive randomisation. The covariates considered were age, weight, and the ASA status of the patients. The groups were protocol group (PG) and control group (CG). The randomisation was carried out by a member of our study team. Two anaesthesiologists (who had not participated in randomisation) conducted the intraoperative management. They performed an intraoperative protocol determined by randomisation. The personnel who conducted postoperative management and postoperative data collection were unaware of how intraoperative management was conducted or the group of patients. The data collected and the patient group were linked after the data collection process was completed.

### Anaesthesia management

Before the procedure, thoracic epidural catheter was inserted in the left lateral position (Th 7–8 or Th 9–10 for rectal surgery) and the tested with 3 ml of 2% lidocaine was performed. After monitoring and placement of the intravenous line, the infusion of dexmedetomidine was started (0.5 mcg/kg/hour). The continuous infusion ended after skin suture at the end of the procedure.

Then a standard induction to general anaesthesia (propofol, sufentanyl, rocuronium) was performed. Anaesthesia was maintained by iv infusion of propofol. The depth of anaesthesia in both groups was adjusted to keep the bispectral index (BIS) 40–55.^25^ Analgesia was provided by 15 ml of 0.25% epidurally levobupivacaine, with a 15 mcg sufentanyl supplement. 1–2 hours after epidural bolus of local anaesthetic, patient-controlled epidural analgesia (PCEA) was started with constant infusion rate and additional patient-controlled boluses for postoperative analgesia (PCEA (0.125% levobupivacaine 200 ml, morphine 4 mg, clonidine 0.075 mg; infusion rate 5 ml/h, bolus 5 ml, lock time 30 minutes). Relaxation was provided with rocuronium and monitored with the train-of-four monitor (TOF). Sugammadex was provided to reverse neuromuscular block at the end of operation.

The haemoglobin level was measured every two hours or at the events with blood loss over 500ml. It was kept above 80 g/L. A fall in haemoglobin was coped with blood transfusion. Oesophageal measured body temperature was kept in the range between 36 and 37 °C.

Postoperatively, the patients were transferred to postoperative recovery and then to Abdominal Surgery high dependency unit (HDU).

### Protocol group

Monitors that calculate stroke volume (SV) and cardiac output (CO) from a standard radial arterial line (LiDCO Rapid, LiDCO Cardiac Sensor Systems, Cambridge, UK) were applied. The near-infrared spectroscopy (NIRS) monitor (INVOS, Medtronic, USA) was used in the protocol group. As a non-invasive technology that continuously monitors regional tissue oxygenation, it was used unilaterally to monitor cerebral oxygenation in the left hemisphere. A baseline prior to induction was recorded. Baseline values of the nominal stroke index (SI), cardiac index (CI), BIS, mean arterial pressure (MAP), and regional oxygen saturation (rSO2) were recorded.

The patients have received 2 ml/kg/h of balanced fluids + replacement for fluid loss (with a ratio of 3 units of balanced crystalloids per every unit of blood loss, until the Hb 80. Then the blood transfusion was started. In the event of immediate blood loss of more than 500 ml, colloids were administered with the ratio to blood loss 1:1. The exact number of fluids given, dependent on monitored hemodynamic variables.

Phenylephrine was administered when the SVV was below 13% of variation, CI was in normal range and there was hypotension. CI, MAP, and SI were maintained within 80% of baseline values. In the event of a 20% fall in regional cerebral oxygenation (rSO2) or values rSO2% below 60 in the absence of a fall in CI or blood loss, we adjusted ventilation so that PaCO2 was kept in the high normal range (5–5,5kPa).

### Control group

The same anesthetic regime was used in PG; there was no hemodynamic monitor. Measurement of cerebral oxygenation was also absent. The patients received 2 ml/kg/h of balanced fluids and additional fluid for replacement for fluid loss as in protocol group. If there were no clinical signs of hypovolemia, phenylephrine was used in treatment of hypotension.

### Data collection

Postoperatively we collected the following data: length of stay, length of stay in HDU, re-admission to HDU or intensive care unit (ICU), quality of wound healing, reoperations, 30-day mortality. We have observed complications (sepsis, pneumonia, acute respiratory infection, pleural effusion, myocardial infarction, pulmonary embolism, stroke, infection).

### Hospital discharge criteria

To reduce unintended variations, strict discharge criteria were implemented. A hemodynamically stable patient without active infection, proper wound healing, and who has completed the first phase of rehabilitation to assisted mobility (or mobility comparable to preoperative) was discharged. If due to administrative reasons formal discharge was not possible, we considered him discharged if all the criteria were met.

### Mental status testing

Preoperative mini mental state test (MMSE) examination was conducted.^26^ The aim was to measure possible postoperative cognitive decline. Postoperatively, the same testing was conducted after patient was admitted to the ward from high dependency unit.

### Statistical analysis

The results were analysed using R: A Language and Environment for Statistical Computing. The results of intraoperative management, the results of postoperative creatinine, the demographics of the patients, and the length of stay are presented as the median and interquartile range. Groups were compared using the Mann-Whitney U test, the level of significance of 0.05 was considered statistically significant.

Intraoperative observations, postoperative complications, and ASA classification are presented as the absolute number of patients with a certain intervention/observation. Groups are compared using the Chi-square test or Fisher's exact test, where appropriate. A level of significance of 0.05 is considered statistically significant.

When comparing postoperative complications, several comparisons are made on the same sample. The level of significance was adjusted accordingly to the Bonferroni correction, and a p-value of 0.001 is considered statistically significant.

## Results

**FIGURE 1.**
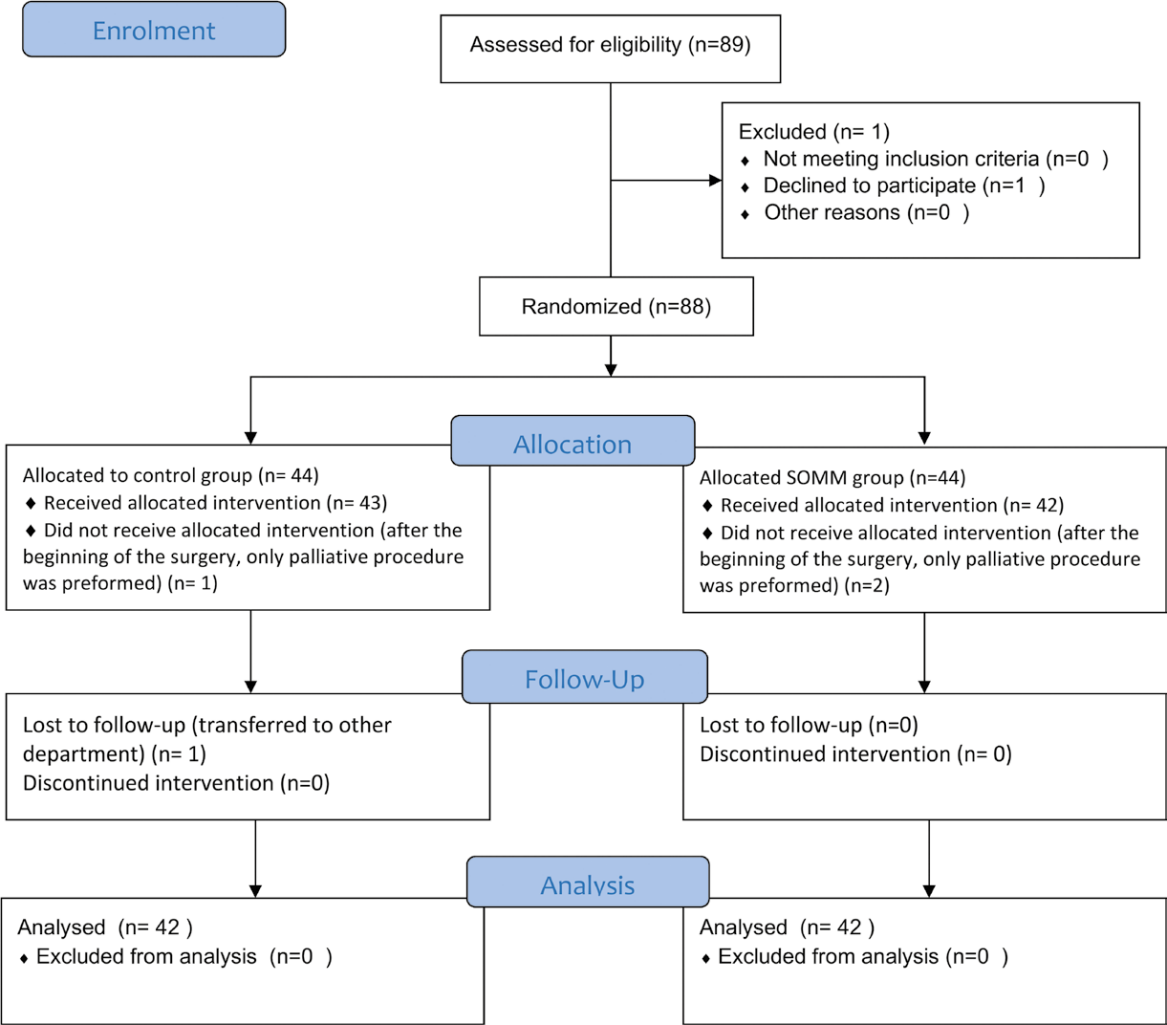
Consort diagram of the study.

We randomly selected 88 patients, 44 in each group. Regarding intraoperative management and postoperative complications, 84 patients were analysed, 4 were excluded after randomisation because the intraoperative protocol was not strictly followed. Consort diagram of the study is shown in Figure 1.

The average age of the included patients was 65 ± 12 years in CG and 66 ± 8 years in PG (P = 0.265, Mann-Whitney U test). The average weight was 64 ± 10 kg in CG and 66 ± 12 kg in PG (p = 0.177, Mann-Whitney U test). 18 patients with ASA 2 status were in CG and 16 in the protocol (p = 0.154, Chi-square test). 24 ASA3 patients were included in CG and 26 in PG (p = 0.117, Chi-square test). The median physiological P-Possum in CG was 21 (interquartile range [IQR] 7) and 20 (IQR 8) in PG (p = 0.322, Mann-Whitney U test). The median operative P-possum was 13 in CG (IQR 5) and 13 (IQR 7) in PG. (p = 0.260, Mann-Whitney U test).

The time of perioperative fasting was 13 ± 2 hours, similar in both groups. The median duration of the surgery (from surgical incision to last suture) is 123 minutes in PG (interquartile range, IQR 35 min), and 120 minutes (IQR 47min) in CG (Mann-Whitney U test, p = 0.157). There was no difference in intraoperative propofol consumption between PG (1.32 g) and CG (1.30 g), (p = 0.860, Mann-Whitney U test.

Table 1 shows intraoperative fluid management in both groups and Table 2 intraoperative interventions with respect to hemodynamic variables.

**TABLE 1.** Intraoperative fluid management

	**Control group Median (interquartile range) ml**	**Protocol group Median (interquartile range) ml**	**P-value (Mann – Whitney U test)**
Intraoperative blood loss	300 (425)	500 (500)	0.182
RBC transfusion	0 (0)	0 (0)	0.185
FFP transfusion	0 (0)	0 (0)	1
Platelet transfusion	0 (0)	0 (0)	0.317
Intraoperative fluid balance	+375 (438)	+1300 (1063)	0.0001
Intraoperative urinary output	205 (100)	300 (200)	0.078

1 = shows statistically significant difference; FFP = fresh frozen plasma; RBC = red blood cell

**TABLE 2.** Intraoperative observations and interventions

	**Control group (Number of patients out of 42 with a certain observation/intervention)**	**Protocol group (Number of patients out of 42 with a certain observation/intervention)**	**P-value**
Bolus of phenylephrine during procedure	31	38	0.043^1^,^2^
Vasoactive support with norepinephrine	1	2	0.500^3^
Mean arterial pressure less than 70mmHg at any time during the procedure	31	36	0.226^2^
Mean arterial pressure less than 50mmHg at any time during the procedure	7	8	0.353^2^

1 statistically significant difference;

2 Pearson Chi-square;

3 Fisher's exact test

In PG, we have observed the results of the NIRS monitor. In 7 cases (out of 42), there was a decrease of more than 20% of the preoperative value during the procedure. The absolute value was never below 45%.

None of the intraoperative interventions influenced hospital or high-response unit (HDU) stay as shown in Table 3.

**TABLE 3.** Comparison of length of stay

	**Control group Median (Interquartile range) days**	**Protocol group Median (Interquartile range) days**	**P-value (Mann – Whitney U test)**
Longest stay in the hospital	9 (8)	9 (5.5)	0.851
Duration of stay in the HDU	4 (3)	3 (1.3)	0.122

HDU = high-depense unit

One person (in CG) died during hospitalisation. Several postoperative complications were observed, the distribution among groups was the same as shown in Table 4.

**TABLE 4.** List of postoperative complications in the first three days after the procedure in both groups

**Postoperative complication/intervention**	**Control group (Number of patients out of 42 with a certain observation/intervention)**	**Protocol group (Number of patients out of 42 with a certain observation/intervention)**	Value P^*^
Readmission to the HDU	5	1	0.136^1^
Admission to the ICU	2	0	0.247^2^
Revision surgery	6	0	0.026^1^
The patient has died before discharge	1	0	0.500^2^
Complications related to the operative procedure (dehiscence, inflammation) first day after the procedure	0	2	0.494^2^
Complications related to the operative procedure (dehiscence, inflammation) third day after the procedure	3	2	1^2^
RBC transfusion needed on the first day after the procedure	2	0	0.513^2^
RBC transfusion required the second or third day after the procedure.	1	2	0.500^2^
Acute kidney disease	3	4	0,500^2^
Troponin leak	0	3	0,241^2^
Median level of C-reactive protein (difference between highest postoperative level in 3 days and preoperative level) Laboratory reference range (0–5 mcg/L)	125 (118)	115 (122)	0.106^3^
Median level of procalcitonin (highest postoperative value in the first 3 days) Laboratory reference range (0–0.50 mcg/L)	0.75 (3.19)	0.3 (0.88)	0.001^3^

Due to multiple comparisons, the significance of the p-value was adjusted accordingly to the Bonferroni correction (significant p value for the variables in the table was < 0.001);

1 Pearson's Chi-square;

2 Fisher's exact test;

3 independent samples Mann-Whitney U test

HDU = high-depence unit; ICU = intensive care unit; RBC = red blood cell

The results of the Mini mental state examination are shown in Figure 1. There were no differences between groups, neither was the postoperative result significantly different.

## Discussion

Findings of our study do not support the benefit of goal directed fluid therapy and cerebral oxygenation monitoring during surgery. There was no decrease the incidence of postoperative complications or duration of hospital stay. However, as opposed to some other studies, our groups were homogenous in terms of surgical procedure anaesthetic and pain management. If those factors are optimised, the contribution of multimodal monitoring seems to be lower than anticipated. Nevertheless, there were some important differences in fluid and vasopressor management among the groups.

### Changes in intraoperative management

The use of multimodal monitoring resulted in differences in intraoperative management. The amount of fluid infused was higher in PG and vasoactive drugs were used more often. That suggests a trend towards more dynamic microcirculation. The most noticeable change in the postoperative period (related to differences in operative management) is a significant difference in the level of procalcitonin. Detailed discussion of those conclusions is provided below.

### Fluid optimisation strategy

The results of the number of fluids given during the surgical procedure present an unexpectedly high fluid load in our PG. This group has received almost twice the number of fluids given in the CG. Intraoperative blood loss is comparable, and PG has a large positive intraoperative fluid balance. Thacker *et al.,* reports the relation between higher fluid load and longer stay.^27^ However, the length of stay was similar in both groups in our study. The choice of fluid (colloides or crystalloides) does not seem to have impact on overall morbidity.^28^

One study compared goal-directed therapy (GDT) with standard fluid therapy in cytoreductive surgery (CRS) with hyperthermic intraperitoneal chemotherapy (HIPEC). The study found that the use of a fluid therapy protocol combined with GDT was associated with a significant reduction in morbidity, length of hospital stay, and mortality compared to standard fluid therapy.^29^ Similarly, Yu *et al*. conducted a controlled before-and-after study to evaluate the benefits of intraoperative goal-directed fluid therapy in major gynaecologic oncology surgery. The study found that the implementation of goal-directed fluid management was associated with a reduced risk of postoperative morbidities, particularly surgical site infections.^30^ Another study analysed the impact of intraoperative fluid balance during pancreatoduodenectomy on the development of postoperative pancreatic fistula (POPF). The study found that fluid balance was significantly associated with the development of POPF, highlighting the importance of appropriate fluid management in pancreatic surgery.^31^

In addition, questions are raised about what the optimal goals of hemodynamic parameters (healthy population-derived normal values, preoperative values, maximal values) should be. Studies have shown that optimising cardiac output and oxygen delivery to higher values intraoperatively (supranormal) did not affect postoperative complications rate, intensive care unit stay, or hospital stay length.^32, 33, 34^ The question of fluid concentration has also been raised. The liberal approach can lead to oedema of the intestines and other tissues that may be responsible for poor tissue healing and other complications.^35^ In abdominal surgery protocol-based fluid restriction reduced the incidence of perioperative complications such as cardiopulmonary events and altered intestinal motility while improving wound and anastomotic healing and reducing hospital stay compared to liberal fluid management.^9,10^ One of the trials has shown a 52% lower rate of major postoperative complications in the restrictive group than in the conventional group.^11^

Our study presents opposite results where the optimised group received a larger number of fluids. Our protocol has clearly defined steps when to add inotropes or fluid. One reason for the fluid load would be vasodilation due to epidural analgesia (all patients in our study have epidural analgesia), although vasoconstrictor (phenylephrine) in boluses was predicted to counteract the effect.^36^ When comparing studies, 60 – 80% of the included patients have epidural analgesia.^21,37^ Lopes *et al.,* reports a significant decrease in ICU and hospital stay in the intervention group with an even greater difference in the number of infused crystalloids and colloids. They report a total volume of fluids infused 7 ml/kg /h in CG (roughly the same as in our study) and 21 ml/kg/h in the intervention group (12,5 in our study).^38^ The choice of fluids and their timing also differs greatly.^20^ The optimised group has received a larger volume of fluids, especially, although not exclusively, colloids.^39^ Rare studies report other factors that greatly affect patient fluid status at the beginning, for example how long prior to the procedure are fasted, are fast track protocols implemented, etc. When trying to explain why sometimes one fluid regime (for example, restrictive) improves the outcome for the most, but not for all, we must realise that instead of restrictive or liberal there is only patient-directed fluid regime. Every patient should receive as much fluid as needed and at an appropriate time.^40^

### Differences in the use of vasopressors

Significantly more patients with PG require vasopressor support with phenylephrine. Some articles suggest that anaesthesia after induction also causes venodilatation and not only arteriolar vasodilation (and consequently the decrease in MAP due to a change in volume out of the arterial tree into the dilated venous compartment).^41, 42, 43^ Phenylephrine infusion before induction minimises this effect, but this is hardly the complete explanation of the difference. To keep hemodynamic parameters as close to starting values as possible, the anaesthetist in PG probably reacted earlier than in CG. In CG, if MAP was kept to some extent (due to reflex mechanisms) there was no information about hemodynamic changes that would require intervention (fall in cardiac output and stroke index). The number of MAP falls below 70 mmHg and 50 mmHg is similar in both groups, but that does not mean that the duration of hypotension is the same.

### Monitoring depth of anaesthesia

BIS was used in both groups. This is in accordance with hospital policy, as total intravenous infusion was used to prevent intraoperative awareness.^44^ In the context of multimodal monitoring, we omit an important variable that, without doubt, influences the outcome. Probably not only cognitive decline, but mortality and morbidity in general are related to too deep anaesthesia, a common occurrence without monitoring, especially in elderly people.^25,45, 46, 47^

### The role of cerebral oximetry

Our study does not confirm the benefit of using a cerebral oximetry monitor during major abdominal surgery, at least it does not influence the results as presented in this study. Cerebral oxygenation monitoring cannot be considered as a monitor of overall tissue oxygenation.^48^ The incidence of renal impairment can be considered one of the measures of adequate oxygenation.

### Postoperative complications and length of stay

Neither the length of stay in the HDU nor the hospital stay decrease in PG. There are some postoperative complications such as the need for revision surgery, indication for antibiotic treatment third day after the procedure, or readmission to HDU that occur largely in the CG. Only comparison of individual complication does not show a statistically significant difference, but if we sum up all three, there is an obvious and statistically significant difference. Some other studies report more convincing but similar results.^49^

C-reactive protein after surgery was elevated in both groups. There were no significant differences between the groups. A significant difference in the highest postoperative levels (in first 3 days) of procalcitonin was noted. Despite this, no clinically or microbiologically evident bacterial infection was confirmed. The level of procalcitonin increases in response to a pro-inflammatory stimulus, especially of bacterial origin.^50^ The median value in CG is above the reference range. Since the surgical management in both groups was similar, different fluid status might explain stronger inflammatory response to tissue trauma in one group. It is important to note that procalcitonin is not a specific marker for bacterial infections and can be influenced by various factors, including noninfectious inflammatory reactions and tissue trauma.^51,52^ Therefore, the role of procalcitonin in noninfectious tissue trauma is still not well-defined, and further research is needed to determine its diagnostic utility in this context.^53^

Troponin leak was observed in 3 patients with PG. The increase is only marginally above the laboratory threshold value for positive. Acute myocardial infarction was ruled out in those patients with a high degree of confidence. Anyway, this can be related to a higher fluid load in the PG.

### Mini Mental State Examination Testing

No major short-term differences in cognitive function are seen. Cognitive changes, related to the anaesthesia are much more subtile.^54, 55, 56, 57, 58^

### Strengths and limitations of the study

The patients involved in the study are very homogeneous in terms of surgical procedures, perioperative surgical management, and comorbidities. Demographic characteristics of both groups were similar. The type of anaesthesia was the same (total intravenous anesthesia [TIVA] with propofol and epidural analgesia) in all the patients observed. Compared to other prospective studies, the number of patients included is comparable.^59^

Multimodal monitoring would probably provide more benefit, if used throughout the entire HDU stay not only during the surgical procedure.

## Conclusions

In the present study, the joint use of hemodynamic monitoring and cerebral monitoring does not significantly decrease the length of stay in HDU or hospital stay in cancer patients after abdominal tumor resection. There is a difference in the volume of fluids infused, that is larger in the protocol group. There is also significantly higher use of vasopressors in the protocol group. The median postoperative value of procalcitonin was significantly higher in control group, suggesting differences in immune response to tissue trauma in different intraoperative fluid status.

There were no significant differences in the number of other postoperative complications observed in the postoperative period. The use of expensive additional monitoring may not provide benefit when used in general abdominal cancer surgery.
